# Niraparib PBPK modeling to predict optimal dosage in patients with hepatic impairment

**DOI:** 10.3389/fphar.2026.1736762

**Published:** 2026-05-11

**Authors:** Anatoly Pokladyuk, Veronika Voronova, Gabriel Helmlinger, Kirill Peskov, Yuri Kosinsky

**Affiliations:** 1 Sirius University of Science and Technology, Sirius, Russia; 2 Modeling & Simulation Decisions FZ-LLC, Dubai, United Arab Emirates; 3 Quantitative Medicines, Boston MA, United States; 4 Research Center of Model-Informed Drug Development, I.M. Sechenov First Moscow State Medical University, Moscow, Russia; 5 Marchuk Institute of Numerical Mathematics of the Russian Academy of Sciences (INM RAS), Moscow, Russia

**Keywords:** ABC-SMC, hepatic impairment, niraparib, PARP inhibitors, PBPK modeling

## Abstract

**Objective:**

To develop a physiologically based pharmacokinetic (PBPK) model of niraparib, predictive of optimal dosage in patients with hepatic impairment and of key pharmacological factors of toxicity.

**Methods:**

A comprehensive systematic literature review was conducted in PubMed and ClinicalTrials.gov, to gather all sources reporting niraparib PK data, in accordance with PRISMA guidelines. The PBPK model of niraparib was built using the PK-Sim® software (version 12.1). Parameter calibration was performed using an Approximate Bayesian Computation Sequential Monte Carlo (ABC-SMC) approach, which was implemented in R (version 4.0.2). Model development, analysis, validation, and forward simulations were performed using the Open Systems Pharmacology (OSP) suite (version 12.3.1) in R.

**Results:**

Five drug-specific parameters (LogP, pKa, intestinal permeability, renal clearance, and carboxylesterase 1 (CES1) metabolism specific clearance (CL_spec_)) were optimized to reproduce plasma concentration–time profiles across dose levels, consistent with mass balance data. Validation against independent datasets confirmed model performance. Hepatic impairment was defined by NCI-ODWG criteria, with CES1 CL_spec_ modeled as a power function of total bilirubin (TBIL) in plasma, to capture impaired clearance. PK simulations under a 300 mg QD regimen across four hepatic impairment groups categorized by μM values of TBIL (mild, moderate, severe60, severe120) demonstrated an increase in steady-state niraparib total concentration in plasma AUCs vs. the predicted normal value of 4155 μM·min, by 33%, 57%, 71% and 101%. Upon dose adjustments to 250 mg for mild, 200 mg for moderate, and 150 mg for the severe60 and severe120 categories, simulations predicted mean AUCs which remained within a ±20% range for all compartments, as compared to patients with normal hepatic function receiving the full dose of 300 mg. A sensitivity analysis showed that pKa and CL_spec_ were the strongest determinants of exposure in the tissue intracellular compartments.

**Conclusion:**

The PBPK model reliably reproduced clinical pharmacokinetics, supported dose adjustment strategies for hepatic impairment, and provides a framework for exploring niraparib safety and further dosing in special populations.

## 1 Introduction

Niraparib is an orally administered poly (ADP-ribose) polymerase (PARP) inhibitor that has shown significant efficacy in the treatment of certain types of cancers (Lee, 2021). It was approved by the U.S. Food and Drug Administration (FDA) in March 2017 for use as a maintenance therapy in patients with recurrent epithelial ovarian, fallopian tube, or primary peritoneal cancer who are in complete or partial response to platinum-based chemotherapy (GlaxoSmithKline, 2023). Despite its efficacy, niraparib is associated with several adverse effects, including hematological toxicities such as thrombocytopenia, anemia, and neutropenia (Shu et al., 2022). These toxicities, along with other factors such as hepatic and renal function, age, and body weight, can significantly influence the safety and therapeutic outcome of the drug. Therefore, a comprehensive understanding of the pharmacokinetics (PK) of niraparib is essential for optimal dose finding in special populations.

Niraparib is primarily metabolized by carboxylesterases (CESs), a class of enzymes which is expressed predominantly in liver and intestinal cells. Lewis et al. (2026) further established that only the carboxylesterase 1 (CES1) isoform (and neither CES2, nor CYP3A4, as suggested earlier) can metabolize niraparib. The resulting inactive metabolites, as well as a portion of the unmetabolized drug are eliminated through renal and hepatobiliary routes (van Andel et al., 2017). Thus, niraparib PK might differ for patients with normal vs. impaired liver function. While clinical studies have investigated exposure of a single-dose niraparib in patients with moderate hepatic impairment, the effect of severe hepatic dysfunction remain insufficiently characterized (Akce et al., 2021). One objective way to extrapolate existing dosing strategies to specific subpopulations, without conducting new clinical studies, is the use of physiologically based pharmacokinetic (PBPK) modeling. This technique is routinely used at different stages of drug development and has proven its utility in enhancing life-cycle management activities and various drug label updates, especially those related to pharmacokinetic investigations (Rowland et al., 2011).

In the current study, a novel PBPK model of niraparib was developed to explore the drug pharmacokinetics in special populations, based on currently available clinical data. PBPK modeling allows to predict drug distribution and exposures across various organs and tissues and can be used in assessing both efficacy and toxicity challenges. In particular, model-based estimations of niraparib concentrations in bone tissue is of interest, due to the drug potential in hematological toxicity. Within the developed PBPK model, a dependence was established between liver function, represented as total bilirubin (TBIL) in plasma, and niraparib specific clearance via CES1 metabolism. Based on this relationship, dosing recommendations were proposed for patients with varying degrees of hepatic impairment, with the goal of achieving exposure levels comparable to those in patients with normal hepatic function.

## 2 Materials and methods

### 2.1 Data

A systematic literature review was conducted in PubMed and ClinicalTrials.gov, to gather all sources reporting niraparib PK data, with a focus on plasma drug concentrations measured in Phase 1 and Phase 2 clinical trials as well as studies of populations with impaired renal or hepatic function. The review adhered to the Preferred Reporting Items for Systematic Reviews and Meta-Analyses (PRISMA) guidelines, ensuring a thorough and transparent approach to data collection (Moher et al., 2009). The search queries were (niraparib) AND ((Clinical Trial[Publication Type]) OR renal OR kidney OR hepatic OR liver) for PubMed and Niraparib AND Completed AND (Phase 1 or Phase 2) for ClinicalTrials.gov. Additionally, studies from pharmacokinetics related sections of the EMA Assessment Report and the FDA Clinical Pharmacology Review (European Medicines Agency EMA, 2020) were examined for the presence of relevant PK data. Articles were screened for eligibility based on abstracts and outcome measures from corresponding clinical trials. Screening results across different sources were merged and duplicates were removed. The resulting list of identified literature sources was additionally investigated to identify references which could contain additional sources of relevant PK data. Thereafter, all pertinent data were digitized and included in an extended database. Two investigators (AP and VV) independently performed searches and corresponding assessments, based on this systematic review process.

### 2.2 PBPK modeling

#### 2.2.1 Niraparib PBPK model development

The full PBPK model consisted of a multi-compartment structure described with system- and drug-related parameters (Rowland et al., 2011). A ‘middle-out’, fit-for-purpose PBPK model was developed for niraparib (Shebley et al., 2018); model structure and system parameters were taken as implemented in PK-Sim, while drug-specific parameters were derived from published sources or estimated based on existing clinical niraparib exposure data following capsule administration in clinical trials. Niraparib is a small molecule compound with a molecular weight of 320.4 g/mol; the fraction unbound (fu) in human plasma is 0.17 (Food and Drug Administration FDA, 2017; European Medicines Agency EMA, 2020). Partition coefficients and cellular permeabilities were calculated using, respectively, the Schmitt and Charge-dependent Schmitt methods implemented in PK-Sim (Schmitt, 2008). An assumption was made about absorption parameters, to simplify the model calibration procedure: niraparib capsule dissolution in the gastrointestinal tract was described using a Weibull function, with a dissolution time of 0.5 h and a dissolution shape parameter of 2.0, based on the duration of a zero-order drug release estimate under fasted conditions (0.235 h or longer) taken from the population PK model (Monk et al., 2024). Relative abundances of the niraparib metabolizing enzyme isoform CES1 in liver, intestine, kidney, heart and lungs were calculated from (Basit et al., 2020) and implemented in the model (calculations are given in Supplementary Table S1). For all other system parameters, default values were used, as reported for full PBPK models (see the PK-Sim project file *niraparibPBPK.pksim5* in the Supplementary Material).

#### 2.2.2 Model parameters: calibration

Five drug-specific parameters, namely, LogP (logarithm of octanol:water partition coefficient; a measure of lipophilicity), pKa (a base compound ionization constant), P_int_ (intestinal permeability; dm/min), CL_ren_ (renal plasma clearance; ml/min/kg) and CL_spec_ (drug specific clearance normalized to the CES1 enzyme concentration; L/min/µmol) were chosen for calibration. An iterative Approximate Bayesian Computation Sequential Monte Carlo (ABC-SMC) approach was used (Beaumont et al., 2009). The ABC-SMC algorithm sequentially refines the parameter posterior distribution approximations to be used, to generate a parameter set for further steps. At each iteration, indexed by t, the algorithm aims to generate draws from p(θ|ρ(Y, Y_obs_) < $\varepsilon_{t}$ ), where $\varepsilon_{t}$ defines a series of decreasing thresholds. ρ(Y, Y_obs_) is the chosen measure of discrepancy between model-predicted (Y) and observed (Y_obs_) concentrations of the drug, i.e., the objective function. In the first iteration, parameter values were sampled from prior distributions, which were assumed to be normal and relatively wide. Mean values for prior distributions of P_int_, CL_ren_, CL_spec_ were primarily selected in the PK-Sim GUI, and theoretical estimates of niraparib logP = 2.45 and pKa = 10 were taken from the DrugBank database (Knox et al., 2024). A relatively large CV = 100% was used for the prior distributions of P_int_, CL_ren_, CL_spec_, and smaller CV = 20% for the logP and pKa prior distributions. Starting from the second iteration, new parameter sets from the parameter space were generated by applying a multi-variate normal (MVN) perturbation kernel to the solutions obtained in the previous iteration (Filippi et al., 2013). The parameters of the MVN distribution (means and covariance matrix) were estimated based on the posterior distribution set obtained in the previous iteration.

The following form of an objective function was taken, to obtain the comparable impacts of relatively low and high observed values:

$$\rho \left(Y,Y_{obs}\right)=\frac{1}{m}\sum_{j=1}^{m}{\left(\ln\left(\frac{y_{j}}{y_{obs,j}}\right)\right)}^{2}$$

Information on unchanged niraparib excretion in urine (van Andel et al., 2017) was integrated into a rejection procedure, to decrease the uncertainty in fitted parameters and to ensure model compatibility with existing published PK data. A proposal was accepted if the predicted amount of drug in urine was within the expected value, plus or minus a 40% interval.

At each iteration, the same threshold value $\varepsilon_{t}$ was used for the $\rho \left(Y,Y_{obs}\right)$ values calculated for different doses of niraparib (if more than one dose was considered). Thus, a proposal was accepted only if it properly described the data from different doses simultaneously. Details of the algorithm realizations are presented in the Supplementary Table S2.

#### 2.2.3 Integration of liver function dependence

The hepatic function of patients is classified based on National Cancer Institute-Organ Dysfunction Working Group (NCI-ODWG) criteria, which were used in the only one clinical trial of niraparib PK featuring patients with normal hepatic function (NHF) and moderate hepatic impairment (MHI) (Akce et al., 2021). According to these criteria, the severity of liver dysfunction is determined by a combination of total bilirubin (TBIL) and aspartate aminotransferase (AST) levels in plasma. The following limits were used to define reference values for hepatic impairment definition: the NHF and MHI groups satisfied, respectively, TBIL ≤ upper limit of normal (ULN) and 1.5*ULN ≤ TBIL ≤ 3*ULN conditions.

In order to integrate the dependence of niraparib clearance on hepatic impairment severity, the following assumption was made: the CL_spec_ parameter of CES1 metabolism was taken as a function of TBIL (Li et al., 2024):

$$CL_{spec}\left(TBIL\right)={CL}_{spec;median}\times {\left(\frac{TBIL}{TBIL_{median}}\right)}^{-\alpha }$$

where ${CL}_{spec;median}$ represents the calibrated parameter value in patients with normal hepatic function and $TBIL_{median}$ corresponds to the median total bilirubin concentration (6.8 µM) derived from the study cohort characteristics. To estimate CL_spec_ in the moderate hepatic impairment (MHI) group, the same calibration algorithm was applied, substituting the MHI group median TBIL value (41.9 µM) into the equation. Parameter α was subsequently determined by solving the equation using the ratio of the MHI and NHF group median TBIL values.

To validate model predictions, additional simulations were made for specific individuals with mild, moderate, or severe grade of hepatic impairment, according to the Child-Pugh criterion (Verbeeck, 2008). The modifications of several physiological parameters (such as functional volume of liver compartment, blood flow through relevant organs, fraction unbound in plasma based on decreased albumin levels, *etc.*, - see full list of parameters in Supplementary Table S3) vs. normal were considered for these individuals, based on studies by (Edginton and Willmann, 2008; Johnson et al., 2010).

Additional simulations incorporated the observed reduction in hepatic CES1 abundance in patients with severe hepatic impairment (Prasad et al., 2018), as detailed in Supplementary Table S1.

#### 2.2.4 Final model analysis

Simulations were performed to predict niraparib plasma exposure changes in patients with differing severity in hepatic impairment and to search for dose adjustment achieving total plasma concentration-time AUCs within the target range predicted in patients with normal hepatic function. Tissue distribution of niraparib, following proposed dose adjustments, was predicted and compared to the comparator NHF group receiving a full dose of the drug.

A local sensitivity analysis was performed to identify model parameters which may drive a higher effect upon drug exposure in physiologically relevant effect compartments, including the bone marrow (as a surrogate for hematological toxicity) and the gonads (a representative site of efficacy, in ovarian cancer subjects). Determination of bone marrow exposures was indeed deemed a priority, due to niraparib dose-limiting hematological toxicities (e.g., thrombocytopenia, anemia), which are mechanistically linked to drug accumulation in hematopoietic tissue. Niraparib exposure in European female patients under a QD regimen with a recommended dose of 300 mg was explored. Values of five (5) model parameters (LogP, pKa, P_int_, CL_ren_, CL_spec_) were independently varied in a range from 85% to 115% relative to final parameter values (Table 1), with a 1.0% step. Steady-state AUC values (AUC_ss_) of niraparib intracellular unbound concentrations in various organs and tissues — per variations of a particular parameter value — were calculated over 24 h, post the 28th daily dose, using the full PBPK model.

**TABLE 1:** Final parameter values.

Parameter	Value (Q1-Q3)	Description	Source
MW (g/mole)	320.4	Molecular weight	Knox et al. (2024)
fu	0.17	Fraction unbound in plasma	Food and Drug Administration (FDA), (2017); European Medicines Agency (EMA), (2020)
logP	2.997	Lipophilicity	calibrated
pKa(base)	9.087 (8.874-9.284)	Base dissociation constant	calibrated
CL_spec_, L/min/μmol	7.49e-03 (6.48-8.76e-03)	Drug specific clearance normalized to the CES1 enzyme concentration (niraparib CES1-dependent metabolism)	calibrated
P_int_, dm/min	6.224e-05 (3.87-8.35e-05)	Intestinal permeability	calibrated
CL_ren_, L/min/kg	3.757e-04 (3.21-4.53e-04)	Renal plasma clearance	calibrated

Q1-Q3, the range from the 25th to the 75th percentile of the posterior.

#### 2.2.5 Software

Concentration-time profiles were digitized using WebPlotDigitizer, version 5.2 (https://automeris.io/wpd/). The core of the niraparib PBPK model was built in PK-Sim® (version 12.1, www.open-systems-pharmacology.org). Data manipulation (packages: dplyr (v. 1.1.4), tidyr (v. 1.3.1), PerformanceAnalytics (v. 2.0.4)) and visualization (packages: ggplot2 (v. 3.5.1), cowplot (v. 1.1.3), ggpubr (v. 0.6.0), patchwork (v. 1.2.0)), model development, analysis and forward simulations (package: ospsuite (v 12.3.1)) were performed using the R software (version 4.0.2, R Project, www.r-project.org).

## 3 Results

### 3.1 Systematic review of available PK data

**FIGURE 1:**
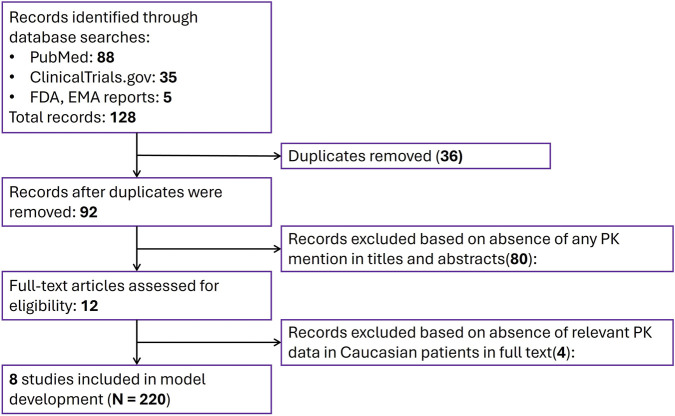
Prisma flow diagram for literature search and selection of clinical trials reporting niraparib PK measurements, for use in PBPK modeling.

Results from the systematic literature review supporting the present PBPK model development are summarized in the PRISM diagram (Figure 1). The initial search of available niraparib PK data resulted in 128 records, 36 of which were removed as duplicates. A total of 80 sources were excluded, based on abstracts and articles which did not include or report any niraparib PK measurements. The remaining 12 articles contained various sets of niraparib PK data and were considered eligible for integration into the final dataset. No studies of niraparib PK in patients with renal impairment were found. One article contained single-dose concentration measurements in patients with moderate hepatic impairment (Akce et al., 2021).

The eight (8) studies selected for model development included relevant PK data in Caucasian patients. In total, aggregated data from 220 patients in 26 clinical trial study cohorts, with 13 different dosing regimens (30, 40, 60, 80, 110, 150, 210, 290, 300, 400 mg QD, as well as 100, 200, 300 SD) were considered. A summary on demographics from the corresponding clinical trials is provided in Supplementary Table S4.

All PK data were obtained in aggregated form, with concentration values reported as either mean or median for each cohort. Niraparib plasma concentration measurements from a Phase 1 multiple ascending dose escalation trial and urine excretion data from a human mass balance study were used for model calibration (Sandhu et al., 2013; van Andel et al., 2017). Model validation was conducted based on clinical data from the six (6) remaining clinical studies featuring observed plasma concentration time profiles for various dosing regimens. An effect of liver dysfunction on niraparib PK was implemented in the model, based on single dose PK data in patients with normal and impaired hepatic function (Akce et al., 2021).

The literature search was again carried out on 15 September 2025; results were updated with 37 additional sources, with 9 removed as duplicates and 24 removed with no PK measurements reported. Out of the 4 remaining studies, only 2 contained PK data relevant to Caucasian patients (Falchook et al., 2024; Yu et al., 2024) and were used for model validation.

### 3.2 Model development

A PBPK model template was prepared in PK-Sim, as described in the METHODS; model structure and initial parameter values were chosen based on available prior information on niraparib pharmacology. The first step in model calibration was to optimize values for the following five drug-specific parameters (LogP, pKa, P_int_, CL_ren_, and CES1 metabolic transformation parameter CL_spec_), to describe niraparib total plasma concentration time profiles from the Phase 1 dose escalation study, following a 210 mg QD dosing (Sandhu et al., 2013). An additional criterion was used in this calibration step, namely,: the levels of unchanged drug in urine could not exceed a 40% deviation from the estimation, based on a niraparib human mass balance study (van Andel et al., 2017). The resultant intermediate parameter distributions are presented in Supplementary Figure S1. To avoid high cross-correlations among parameters in posterior distributions, two of the five parameters (LogP and CL_spec_) were then fixed using the median values of the resulting posterior distributions. In a second step, an iterative ABC-SMC procedure was applied to the three remaining parameters (P_int_, CL_ren_, pKa). The resulting posterior distributions for these parameters are presented in Supplementary Figure S2. Table 1 summarizes the final parameters of the model, which were derived from various sources and/or calibrated as described above (Food and Drug Administration FDA, 2017; European Medicines Agency EMA, 2020; Knox et al., 2024).

### 3.3 Diagnostics and validation of the developed PBPK model

**FIGURE 2:**
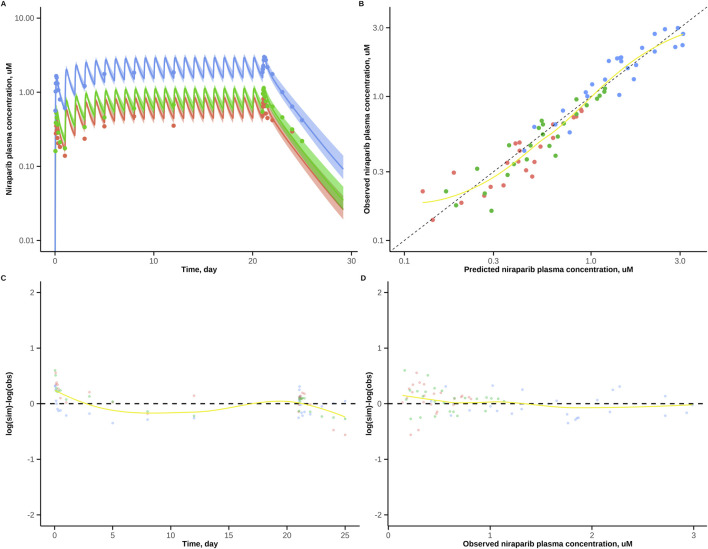
Diagnostic plots for the niraparib PBPK model. Goodness-of-fit plots evaluating model performance against calibration data. **(A)** Simulated median time-concentration profiles (solid lines) overlaid with observed data points. Shaded areas indicate 95% credible intervals. **(B)** Predicted *versus* observed concentrations. **(C,D)** Residual diagnostics plots: Log-scale residuals (log-predicted minus log-observed) as a function of time **(C)** and as a function of observed concentrations **(D)**. In panels **(B–D)**, the yellow curve represents a local regression (LOESS) smooth, indicating the trend of the residuals. Different dosing regimens are color-coded: red (60 mg QD), green (80 mg QD), and blue (210 mg QD).

Publicly available data did not feature individual PK profiles, hence it was not possible to account for inter-individual variability. Consequently, model predictions of typical plasma concentrations for the obtained posterior parameter distributions were used in diagnostic and validation steps. The model adequately described the calibration data; residuals did not demonstrate any functional systematic decline pattern (Figure 2).

**FIGURE 3:**
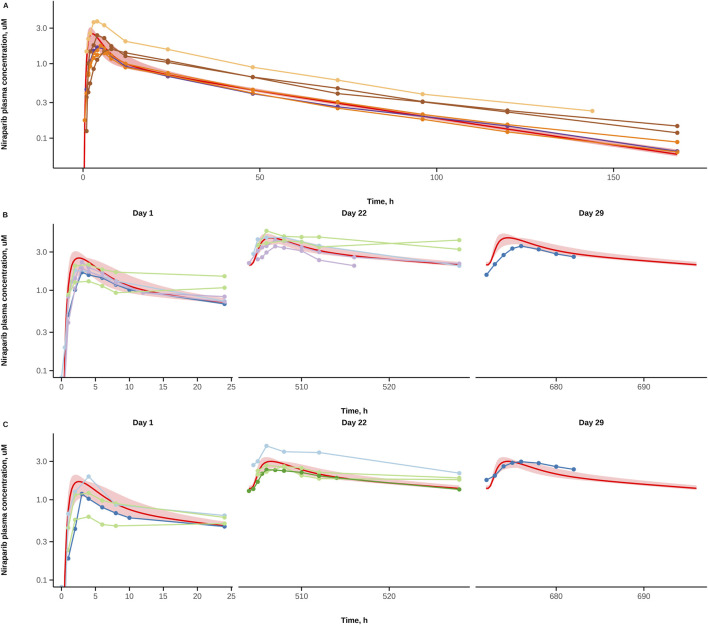
Validation of niraparib PBPK model predictions (red line–model prediction with final parameters, shaded area - 95% CI) against observed clinical data (points) for: **(A)** 300 mg SD, **(B)** 300 mg QD, and **(C)** 200 mg QD regimens. Data points are from clinical trials (Akce et al., 2021; Moore et al., 2018; van Andel et al., 2018; Falchook et al., 2024) **(A)**, (Mirza et al., 2019; Saad et al., 2021; Yap et al., 2022) **(B,C)**, (Sandhu et al., 2013) [Part A,B: 300 mg QD] **(B)**, (Yu et al., 2024) **(C)**. CI, credible interval.

Niraparib total plasma concentration profiles in European female patients under a single dose or a daily dosing regimen were predicted, to account for parameter variability and to compare it with the available observed data. To estimate the corresponding 95% credible interval, calculations were repeated for each parameter combination, from a final posterior distribution set (n = 328). Simulations with the currently approved 300 mg and 200 mg doses were performed to validate the developed model against independent data not used for model calibration. As shown in Figure 3, the model adequately described niraparib mean concentration-time profiles, under either SD or QD drug regimens. Figure 3A shows a notable discrepancy in T_max_ values between model predictions and observed data; however, it can be explained based on significant between-study variability, as T_max_ following single-dose niraparib administration is reported to occur within 2.5–4.0 h (European Medicines Agency EMA, 2020; GlaxoSmithKline, 2023). In Figure 3B, model simulations demonstrate higher niraparib plasma concentrations on Day 29 (predicted C_max_ value is larger by 33%) than observed data from single clinical study (Saad et al., 2021); there is a similar overprediction vs. the observed data from the same clinical study on Day 1, which is indicative of a minor overprediction across the whole period of drug administration for the same cohort. While incorporating random effects for between-study variability may improve alignment with experimental data, it was beyond the present scope. Nevertheless, the model successfully captured the primary PK properties of niraparib, as further evidenced by the residual plots for the validation dataset (see Supplementary Figure S3).

### 3.4 PBPK model simulations for niraparib exposure in patients with hepatic impairment

Five groups were considered, based on liver dysfunction severity, to explore the potential impact of hepatic impairment on niraparib exposure: (i) normal hepatic function (NHF, TBIL = 6.8 μM); (ii) mild (TBIL = 21.25 μM); (iii) moderate (MHI, TBIL = 41.9 μM); (iv) severe60 (SHI60, TBIL = 60 μM); and (v) severe120 (SHI120, TBIL = 120 μM) hepatic impairment. Calibration of CL_spec_ (specific clearance normalized to the enzyme concentration of niraparib CES1 metabolism) based on the PK data from the MHI group is presented in Supplementary Figure S4. Parameter α , relating CL_spec_ to TBIL dependence, resulted in a calculated value of 0.316. The resulting CL_spec_ dependence on total bilirubin level is presented in Supplementary Figure S5.

**FIGURE 4:**
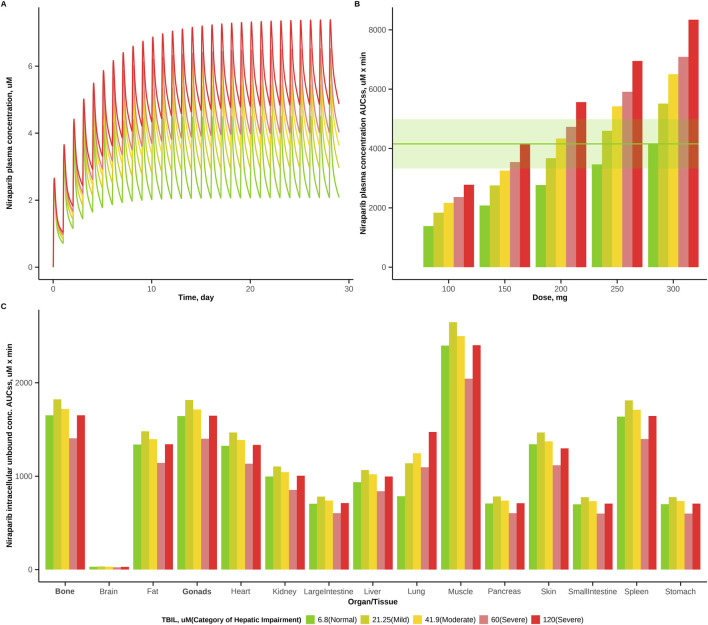
Impact of hepatic impairment on niraparib exposure and model-derived dose adjustments. **(A)** Simulated plasma concentration-time profiles in five hepatic function groups after a 300 mg QD dose. **(B)** Predicted steady-state AUC (AUC_ss_) for each group at various doses - shaded area (light green) indicates the ±20% exposure range in the normal hepatic function (NHF) group following a 300 mg dose. **(C)** Predicted intracellular unbound AUC_ss_ in tissues for each group after dose adjustment (Mild: 250 mg; Moderate: 200 mg; Severe60 and Severe120: 150 mg). TBIL, total bilirubin.

Simulations of niraparib plasma exposure under a QD regimen, for doses in the range of 100 mg–300 mg and with a 50 mg dosing step were conducted to compare niraparib exposures in blood plasma across the five groups and to propose a model-derived dose adjustment, so that changes in predicted plasma concentration AUC at steady-state (AUC_ss_) values in the various hepatic impairment groups vs. the NHF group (with patients receiving the recommended dose of 300 mg) would not exceed 20%. Simulations results are shown in Figure 4.

The model predicted a plasma C_max_ of 4.51 μM for niraparib, under a 300 mg QD regimen in patients with normal hepatic function. For the hepatically impaired groups, C_max_ values increased with liver dysfunction severity: 5.44 μM, 6.12 μM, 6.52 μM and 7.38 μM, respectively (Figure 4A). Similarly, AUC_ss_ in plasma increased, as compared to the predicted ‘normal’ value of 4155 μM·min, by 33%, 57%, 71% and 101%, respectively. Upon dose adjustments to 250 mg for mild, 200 mg for moderate, and 150 mg for the two severe hepatic impairment groups, simulations predicted AUC_ss_ values which remained within a ±20% range of a ‘normal’ exposure level in NHF patients receiving the full 300 mg dose (Figure 4B). Specifically, plasma AUC_ss_ in the MHI group deviated by only 4.4% from that in the NHF group, which supported the recommended dose adjustment of 200 mg for this MHI population (GlaxoSmithKline, 2022).

Niraparib tissue distributions were further evaluated to support the proposed dose adjustments. Predicted intracellular unbound concentration AUC_ss_ values of niraparib in various organs for each of the five groups following dose adjustments are presented in Figure 4C. AUC_ss_ values changed by no more than 20% from the reference value in NHF patients. Also, model simulations showed that the proposed doses preserved the bone-to-gonads ratios in intracellular unbound exposures, a metric which may be indicative of an unchanged toxicity-to-efficacy window in ovarian cancer, one of the key indications of niraparib.

Drug distribution to brain was also evaluated, via simulations of niraparib exposure in this organ, following a 300 mg QD regimen. Intracellular and interstitial unbound, as well as total intracellular concentrations in the brain compartment are presented in Supplementary Figure S6. Despite small intracellular unbound concentration values, niraparib exhibited sustained intracellular drug retention in brain tissue, which may result in higher drug accumulation in hepatically impaired patients. However, the proposed dose adjustments resulted in minimal deviations of niraparib exposure in brain.

### 3.5 Niraparib PBPK model application for individuals with different Child-Pugh hepatic impairment grades

**FIGURE 5:**
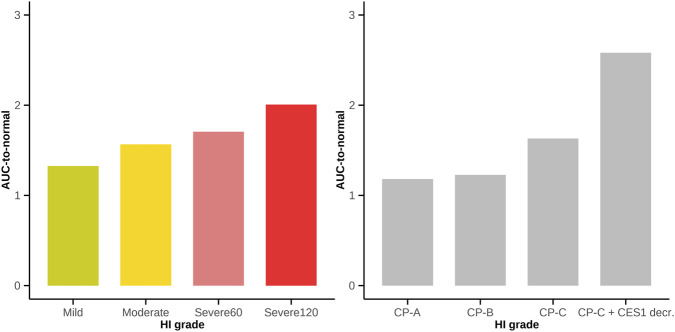
Model-predicted niraparib plasma exposure (300 mg QD AUC_ss_) for various hepatic impairment groups, classified by NCI-ODWG (left) and Child-Pugh (right) criteria, relative to predictions for the group with normal hepatic function.

In order to validate the empirical approach for hepatic impairment extrapolation described above, additional PBPK simulations were performed (Figure 5). A straightforward approach to estimate hepatic impairment effect on drug exposure was to consider prior knowledge on changes in related human physiological parameters, in healthy and hepatically impaired populations. Model simulations were performed in PK-Sim, via integration of specific built-in individuals with various Child-Pugh (CP) grade-specific modifications of physiological parameters (see Supplementary Table S3 for details).

Firstly, the relative CES1 abundances calculated for subjects with NHF were used for individuals with various CP grades. The predicted increase in niraparib 300 mg QD AUC_ss_ for severe (CP-C) hepatic impairment was 164% vs. normal. A similar increase in niraparib exposure, to 171% vs. normal, was predicted by the empirical approach for individuals in the SHI60 group, corresponding to severe (NCI-ODWG) hepatic impairment.

Secondly, the information from (Prasad et al., 2018) on CES1 abundance in liver in patients with end-stage liver disease (∼3.3 fold decrease) was considered in the model. This factor, together with other related physiological parameter changes implemented in PK-Sim and reflecting severe (CP-C) hepatic impairment resulted in an increase in niraparib AUC_ss_ of up to 259% vs. normal. Using the empirical approach, a maximal increase of 201% vs. normal was predicted, for individuals in the SHI120 group. In summary, both hepatic impairment modeling approaches yielded comparable increases in exposure, as a function of impairment grade. However, drug exposures for similar HI grades should not be directly compared across Child-Pugh and NCI-ODWG criteria, due to differences in the underlying liver disease mechanisms of non-cancer vs. cancer populations. Consequently, the NCI-ODWG criterion is more suitable for PK analyses in Oncology patients with hepatic impairment, because of its greater relevance to clinical practice (Lin and D’Cunha, 2018; Elmeliegy et al., 2021). Overall, model predictions obtained by different methods showed consistent results, when considering the discordance observed in patient distributions into hepatic impairment categories, when classified by CP and NCI-ODWG (Elmeliegy et al., 2021).

### 3.6 Exploration and sensitivity analysis of niraparib exposure metrics

**FIGURE 6:**
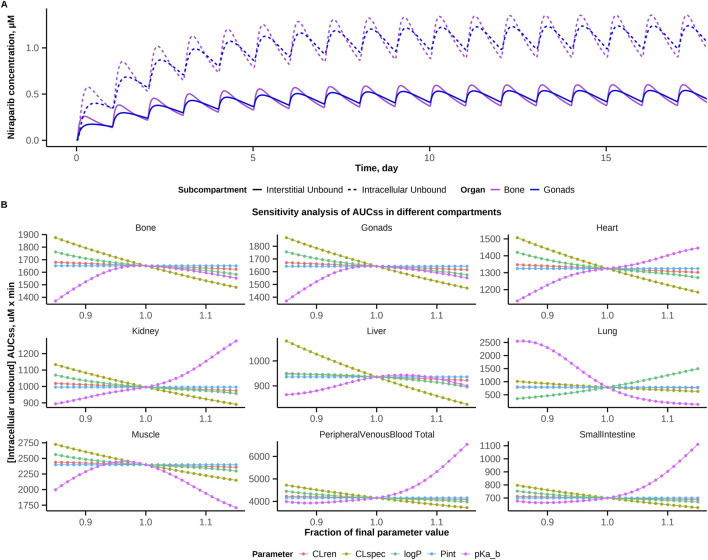
Exploration of niraparib exposure metrics. **(A)** Model-simulated niraparib exposure in bone and gonad compartments. Predicted unbound concentration-time profiles in bone and the gonads under a 300 mg QD regimen. **(B)** Local sensitivity analysis of key model parameters affecting steady-state AUC (AUC_ss_) in bone, the gonads and other organs and tissues.

The developed model was used to explore niraparib exposures in several organ compartments. Model simulations for niraparib intracellular and interstitial unbound concentrations time profiles in bone (as a site susceptible to toxicity) and gonads (as an effect compartment, when treating ovarian cancer) are presented in Figure 6A. The model-predicted steady-state intracellular unbound AUCs in bone and gonad compartments were comparable: 1651.06 μM·min and 1642.80 μM·min, respectively.

A local sensitivity analysis was performed, to identify model parameters which would most affect exposure under a 300 mg QD regimen in different tissues. The profiles of the intracellular unbound concentration AUC_ss_ in various organs and tissues were predicted via simulations, while varying values of five key parameters in a –15% to 15% range (Figure 6B). AUC_ss_ dependence on CL_ren_, CL_spec_, P_int_, logP was nearly linear, while, for pKa, the relationship was more complex and varied across compartments. Specifically, the model pKa parameter value corresponded to a local maximum of intracellular unbound AUC_ss_ in the bone, gonads and muscle compartments, hence any modulation on that value within a 15% range would result in decreased niraparib intracellular exposure. Interestingly, for heart, kidney and lung, a local maximum of the AUC_ss_ was shifted along the pKa-axis relative to the reference value. These shifts can be explained by variations in intracellular pH values in different compartments, as used in the model. CES1 abundances considered in liver, heart, kidney, lung and small intestine compartments also affected AUC_ss_ values. The pKa dependencies of AUC_ss_ in the small intestine and plasma appeared similar, with local minima shifted to smaller pKa values. This may be explained by the drug extensive exchange between these two compartments, whereby the dynamics of drug concentrations in plasma drive the dynamics of intracellular unbound concentrations in the intestine.

## 4 Discussion

PARP inhibitors are associated with significant hematological toxicity: a meta-analysis of 29 randomized controlled trials demonstrated that PARP inhibitors are linked to a significantly increased risk of hematologic adverse events, including all-grade anemia, neutropenia and thrombocytopenia (Wang and Li, 2021). Importantly, niraparib carries a markedly higher risk of grade 3-4 thrombocytopenia, along with an elevated incidence of anemia and neutropenia, as compared to other PARP inhibitors (Valabrega et al., 2021). Therefore, its hematologic toxicity profile requires specific attention in treatment planning, which in turn requires a detailed and quantitative understanding of intrinsic and extrinsic factors potentially affecting exposure to niraparib.

Various niraparib modeling efforts focused on population pharmacokinetics and exposure–response relationships across clinical studies. Notably, one of the studies characterized a three-compartment popPK model, which demonstrated that individualized starting doses (200 mg vs. 300 mg) based on baseline weight and platelet counts could improve safety, particularly in reducing grade ≥3 thrombocytopenia, without compromising efficacy (Monk et al., 2024). Exposure–response analyses further confirmed that higher systemic exposure has been associated with increased adverse events, supporting personalized dosing schemes. Further population PK modeling included a pooled analysis in metastatic castration-resistant prostate cancer; it showed that key covariates such as creatinine clearance and race incurred minimal impact on niraparib exposure, reinforcing the clinical appropriateness of fixed-dose regimens in that setting (Russu et al., 2025). Other studies have explored pharmacokinetics in additional populations, e.g., Chinese and Japanese patients, where niraparib exhibited dose-proportional exposures and favorable tolerability; body weight exhibited modest effects, and no clinically meaningful racial differences were identified (Zhang et al., 2020). Investigations into hepatic and renal impairment via population PK and exposure–response modeling indicated that moderate hepatic impairment increased niraparib exposure, justifying dose reduction to 200 mg, whereas renal impairment had negligible effects (Bruin et al., 2022). However, the above modeling efforts were limited both in scope and in modeling datasets; the latter were used for model calibration, and limitations in model scope did not allow for simulation-based extrapolations to personalized dosing strategies, to benefit a maximal number of eligible patients yet with differences in their clinical profiles. PBPK modeling is a proven quantitative methodology that may significantly expand the scope of drug exposure predictions in multiple compartments of interest, in specific patient populations, while providing a mechanistic basis underlying the simulated dose-exposure profiles; for example, PBPK has been used successfully to rationalize and perform drug dose adjustments in patients with varying degrees of hepatic impairment (Heimbach et al., 2021).

In January 2026, Lewis and coauthors presented a PBPK model of niraparib (Lewis et al., 2026). The model was developed using Simcyp (version 22; Certara, Sheffield, UK). A series of *in vitro* studies were conducted to establish details of niraparib carboxylesterase metabolism, which were later used in the model development. The model was applied to simulate niraparib exposure in cancer patients, including different special populations such as hepatically impaired subjects. Despite predictions being systematically higher than corresponding observed values in the MHI cohort (Akce et al., 2021), plasma exposure metrics aligned well: mean C_max_ was within 1.25-fold and mean AUC within 2-fold of the observed values. However, projections in patient populations with varying degrees of hepatic impairment severity were not presented in that study.

The generalized niraparib PBPK model we developed and validated using PK-Sim® software (version 12.1) enabled a detailed, time-dependent quantification of drug levels in critical effect compartments, including the bone marrow, and may help explain higher rates of niraparib-associated hematological toxicities, including anemia and thrombocytopenia. The incorporation of key niraparib pharmacokinetic properties, along with consideration of different methods to interpret and extrapolate existing hepatic impairment data, provided a framework for a more robust examination of PK in Oncology patients with hepatic impairment, facilitating tailored dose optimization strategies.

Intracellular transformation into a biologically inactive metabolite via CES1 and elimination from plasma via kidney both contribute to the clearance of niraparib; these processes were parameterized based on the available data, including human mass balance data (van Andel et al., 2017). Niraparib is a weak base, with a pKa estimated to be higher than 9.0 (GlaxoSmithKline, 2023; Knox et al., 2024), thus, a major fraction of the drug is positively charged at physiological pH of 7.0–7.4. At the same time, a minor fraction of neutral drug molecules may effectively contribute to overall transmembrane permeability. In the current work, charge-dependent permeability and distribution coefficients were considered in the development of the PBPK model (Schmitt, 2008). Cellular uptake of weak bases is strongly influenced by small differences in intra-vs. extracellular pH. Given a normal pH gradient in healthy tissues (pH of ∼7.4 and 7.0 in extracellular vs. intracellular space, respectively), drugs such as niraparib, with a pKa around 9.0 to 10, may accumulate at higher concentrations intracellularly, as compared to extracellularly. Thus, in various healthy tissues, the model predicted niraparib intracellular unbound concentrations to be ∼two-fold higher than the interstitial (extracellular) unbound concentration. However, when the pH gradient is inverted, such as in cancer cells (pH of ∼6.9 and 7.5 in extracellular vs. intracellular space, respectively), concentrations of weakly basic drugs can be significantly higher extracellularly vs. intracellularly (Webb et al., 2011).

Niraparib is primarily metabolized to an inactive metabolite via carboxylesterases, with minor involvement of CYP450 enzymes. Clinical studies indicated that moderate hepatic impairment may increase the niraparib total plasma concentration AUC by ∼56%, while not significantly affecting C_max_ (Akce et al., 2021). Changes in drug plasma concentrations associated with liver dysfunction may be attributed to several factors, including a decrease in the functional volume of the liver, alterations in hepatic and intestinal blood flow, in the unbound fraction of the drug, and in hepatic enzyme and transporter activity (thereby affecting intrinsic clearance) (European Medicines Agency EMA, 2005; Talal et al., 2017). The very low hepatic extraction ratio of niraparib (van Andel et al., 2018) indicates that its hepatic clearance is predominantly influenced by the unbound fraction and intrinsic clearance, rather than hepatic blood flow. Plasma binding of niraparib did not exceed 90%, and data on the effect of hepatic impairment on this fraction are lacking; hence, the impact of potential changes in plasma protein binding on hepatic clearance remains unclear (Verbeeck, 2008).

In this study, changes in intrinsic hepatic clearance were recapitulated by adjusting the niraparib specific clearance parameter, CL_spec_, of CES1-mediated metabolism as a dependence on TBIL concentration. Calibration of CL_spec_ to describe single dose niraparib plasma concentrations in patients with MHI resulted in good agreement between model simulations and the observed clinical data. The extrapolation of the empirical dependence of CL_spec_ to severe hepatic impairment is not evident and requires further validation, while no information about niraparib exposure for individuals with SHI was available. In a meta-analysis (Mahmood, 2025) of 52 drugs in patients with SHI, the increase in AUC_ss_ vs. normal strongly varied among drugs, in a range of ∼1 to 6 fold. For validation purposes, additional PBPK model simulations were performed using prior information on physiological parameter changes in individuals with various Child-Pugh HI grades. Model-predicted increases in niraparib AUC_ss_ obtained by the two different modeling approaches investigated were consistent with each other.

While the use of a more mechanistic approach to HI in PBPK modeling seems preferable, it is limited by our knowledge on physiological parameters (e.g., abundance of metabolizing enzymes) in individuals with differing liver diseases and toxicities. The suggested empirical approach of CL_spec_ values adjusted according to their relationship with TBIL plasma concentrations may be more generalizable. Since CES1 is most abundant in liver, effective CL_spec_ values strongly correlated with remaining functional volume of liver, portal vein blood flow, and related changes in CES1 abundance in hepatocytes. Thus, the suggested approach was deemed reasonable and applicable for niraparib dose individualization. However, further inference could be made using the model, when accounting for various physiological changes associated with liver dysfunction, especially considering the most severe stages of impairment. For example, changes in the unbound fraction of the drug based on decreasing albumin levels with increasing liver disease severity may be further considered in the model and is supported by previous analyses (Marsousi et al., 2017; Li et al., 2018; Heimbach et al., 2021).

Under the assumptions mentioned above, the developed PBPK model was used to design a tailored dose justification strategy. Specifically, a dose adjustment scheme was proposed for patients with varying degrees of hepatic impairment, classified according to the NCI-ODWG criteria. Patients were stratified based on total bilirubin level, which has been identified as a predominant factor for assessing hepatic dysfunction severity in cancer patients (Patel et al., 2004). Dose adjustments were selected to ensure that model-predicted steady-state AUCs in each physiological compartment remained within ±20% of the AUC predicted for patients with normal hepatic function and receiving the full 300 mg dose. The resulting dosing recommendations aligned well with existing label guidance for patients with MHI (GlaxoSmithKline, 2022), highlighting the utility of PBPK modeling as a rational framework for dose optimization, in the absence of comprehensive clinical data.

A local sensitivity analysis was also conducted to identify key pharmacological parameters influencing niraparib toxicity. Specifically, the analysis focused on the model-predicted intracellular unbound concentration AUCs in bone compartment, used here as a measure of toxicity. Among the tested parameters, physicochemical properties of the drug - pKa and CL_spec_ - were found to exert the greatest influence on this ratio. These results suggest that small changes in molecular properties can substantially alter the tissue exposure profile of niraparib and thus may play a critical role in modulating its toxicity. These findings not only enhance the understanding of the pharmacological behavior of niraparib but also offer valuable insights into rational design of safer, effective, next-generation PARP inhibitors.

Several model limitations should be considered. Firstly, even though niraparib is a substrate of the efflux transporter P-gp, its high passive permeability likely dominates the net flux. Reported efflux ratios for niraparib are 2–5 times lower than for olaparib (Sun et al., 2018). Also, P-gp might contribute to niraparib elimination via the kidneys. Due to limited quantitative data on transporter kinetics to parameterize the effect, P-gp efflux was not explicitly considered in the model; yet it was implicitly considered in the effective P_int_ and CL_ren_ parameter values. Secondly, the K_m_ of niraparib CES1-mediated metabolism could not be precisely determined based on the available data. Clinical PK data are dose-proportional up to 400 mg (Lee, 2021), indicating that niraparib intracellular unbound concentrations (1.0–2.0 μM) are likely to be well below the K_m_ value. Thus, niraparib metabolization on CES1 was considered as an approximate first-order reaction and the specific clearance value, CL_spec_, was subsequently optimized. Thirdly, the model was built using the available published aggregated data, which included niraparib plasma concentrations and mass-balance fractions. Consequently, a detailed analysis of the between-study variability was out of scope in the present work.

An important further extension of this PBPK model may reside in the incorporation of a tumor compartment, e.g., an ovarian cancer tissue compartment, to investigate niraparib distribution in malignant *versus* healthy tissues. Dysregulated pH is recognized as a hallmark of cancer; it has been characterized by a reversed pH gradient in which intracellular pH in cancer cells is constitutively elevated, while the extracellular pH in the tumor interstitial fluid is decreased, as compared to healthy tissue (Helmlinger et al., 1997). Given niraparib is a weak base, its unbound intracellular concentration in a tissue depends on the ratio of intracellular-to-extracellular pH (Webb et al., 2011). By capturing these pathophysiological features in the tumor microenvironment, an extended PBPK model will enable further predictions of niraparib and other PARP inhibitor therapeutic windows, with further detailed simulations of drug concentrations in efficacy and toxic effect compartments.

## 5 Conclusion

In this study, we developed a generalized physiologically-based pharmacokinetic (PBPK) model for the PARP inhibitor drug niraparib, incorporating its physicochemical properties and metabolic pathways into the model. After integration of key processes and parameterization based on the Approximate Bayesian Computation Sequential Monte Carlo algorithm, the model adequately reproduced mean niraparib plasma concentration-time profiles for various drug regimens administered clinically. The model was applied to support the strategy for optimizing dosing regimens in patients with various degrees of hepatic impairment, by integrating clinical data on single-dose niraparib exposure in patients with moderate hepatic impairment and model-predicted drug concentrations in various compartments across specific patient populations. The present modeling research study proposed a methodology strongly anchored into pathophysiology and pharmacology, demonstrating how such a PBPK model can be successfully developed using open access software packages and qualified based on published aggregated clinical data. The PBPK model also offers a unique opportunity for further extensions and hence applications, particularly in the further development of PARP inhibitors in Oncology, including (but not limited to) the enhancement of a PARP inhibitor therapeutic window, applications to specific patient populations, and other post regulatory registration and life cycle management activities.

## Data Availability

The original contributions presented in the study are included in the article/Supplementary Material, further inquiries can be directed to the corresponding author.
