# Supplementary material for: Niraparib PBPK modeling to predict optimal dosage in patients with hepatic impairment
Source: Front Pharmacol. 2026 May 11;17:1736762. doi: 10.3389/fphar.2026.1736762 (PMC13199239; doi:10.3389/fphar.2026.1736762)
Supplement: Supplementary file 1 [file Supplementaryfile1.docx]

Supplementary Material

Table of Contents

[1 Supplementary Figures 2](#_Toc225160277)

[1.1 Supplementary Figure S1: Intermediate posterior distributions and characteristics of parameters after the first step in model calibration 2](#_Toc225160278)

[1.2 Supplementary Figure S2: Final posterior distributions and characteristics of parameters after the second step in model calibration 3](#_Toc225160279)

[1.3 Supplementary Figure S3: Residuals, Validation data 3](#_Toc225160280)

[1.4 Supplementary Figure S4: CL_spec_ parameter optimization describing the observed niraparib plasma PK data, MHI group 4](#_Toc225160281)

[1.5 Supplementary Figure S5: Hepatic metabolism parameter CL_spec_ (1/min/μM of enzyme) dependence on total bilirubin (TBIL) level 4](#_Toc225160282)

[1.6 Supplementary Figure S6: Model-predicted niraparib concentrations in brain compartment 5](#_Toc225160283)

[1.7 Supplementary Figure S7: Model-predicted niraparib plasma PK profiles for different hepatic impairment grades 5](#_Toc225160284)

[2 Supplementary Tables 6](#_Toc225160285)

[2.1 Supplementary Table S1: Relative CES1 abundances in various tissues 6](#_Toc225160286)

[2.2 Supplementary Table S2: Summary of Approximate Bayesian Computation Sequential Monte Carlo (ABC-SMC) algorithm parameters 7](#_Toc225160287)

[2.3 Supplementary Table S3: Physiological parameter values used in PK-Sim niraparib PBPK model for patients with hepatic impairment 8](#_Toc225160288)

[2.4 Supplementary Table S4: Summary of demographics in clinical trials featuring niraparib PK 9](#_Toc225160289)

# Supplementary Figures

## Supplementary Figure S1: Intermediate posterior distributions and characteristics of parameters after the first step in model calibration


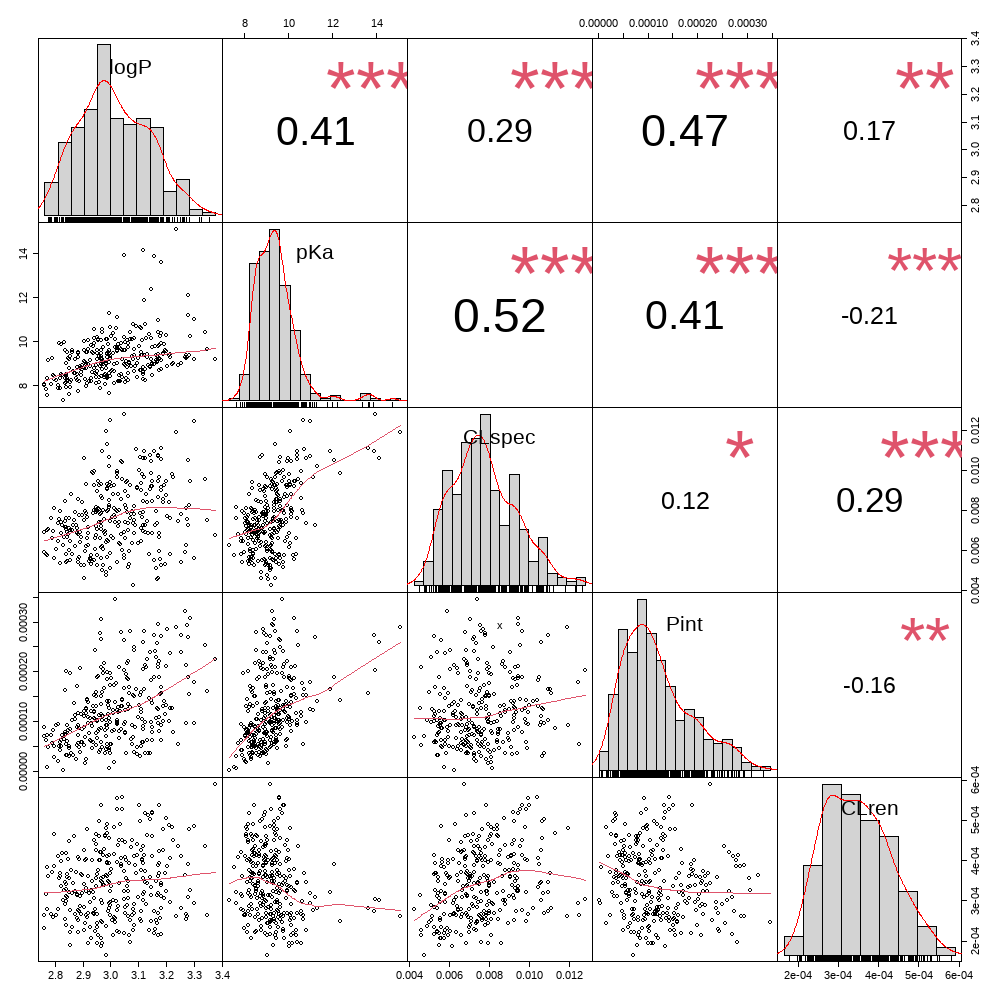
Pearson correlation coefficients are shown. Significance levels are based on two-sided tests: * p < 0.05, ** p < 0.01, *** p < 0.001.

## Supplementary Figure S2: Final posterior distributions and characteristics of parameters after the second step in model calibration


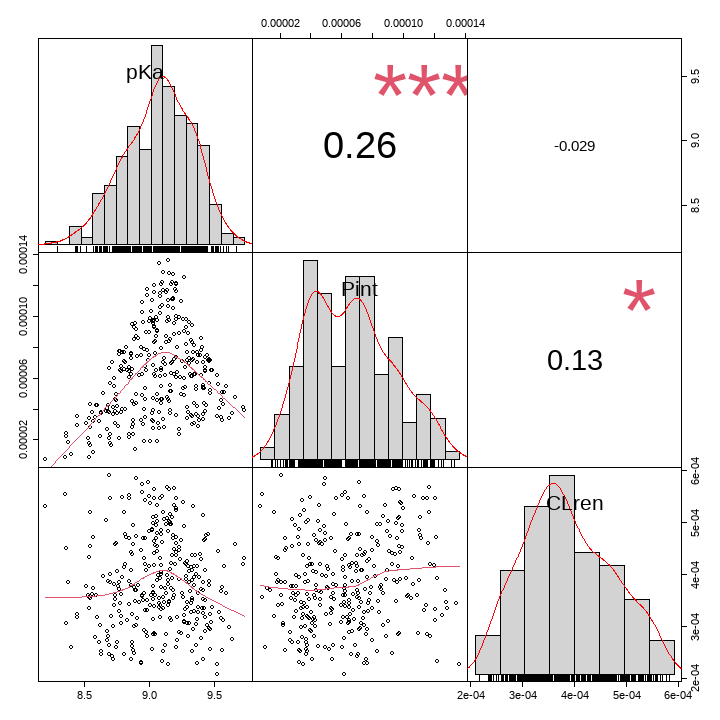


Pearson correlation coefficients are shown. Significance levels are based on two-sided tests: * p < 0.05, ** p < 0.01, *** p < 0.001.

## Supplementary Figure S3: Residuals, Validation data


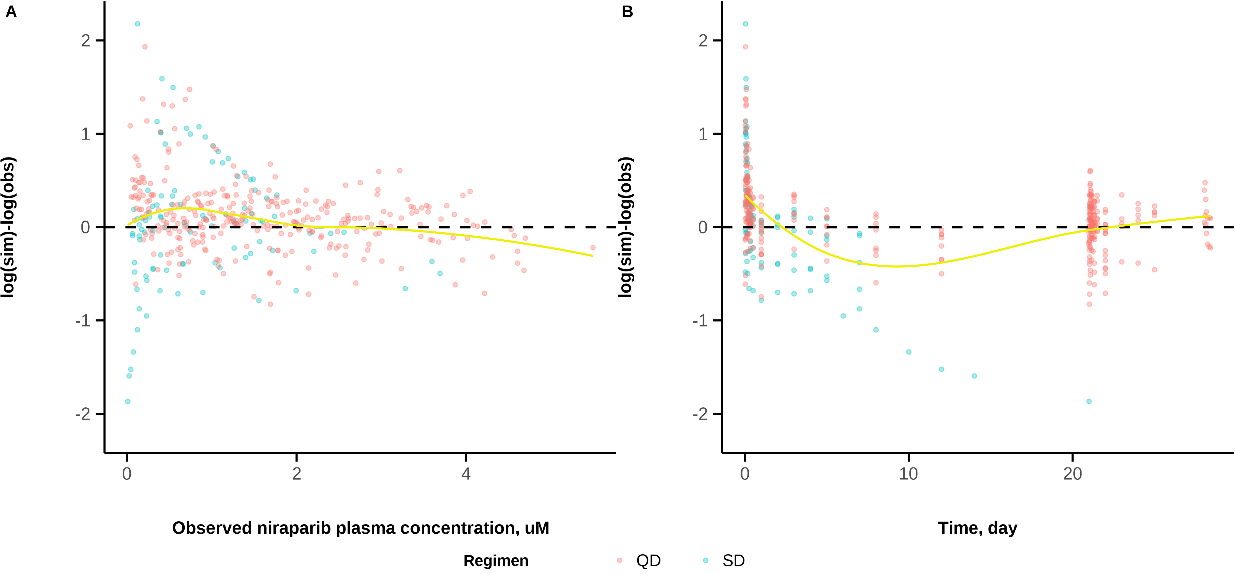


Yellow curve – local regression (LOESS).

## Supplementary Figure S4: CL_spec_ parameter optimization describing the observed niraparib plasma PK data, MHI group


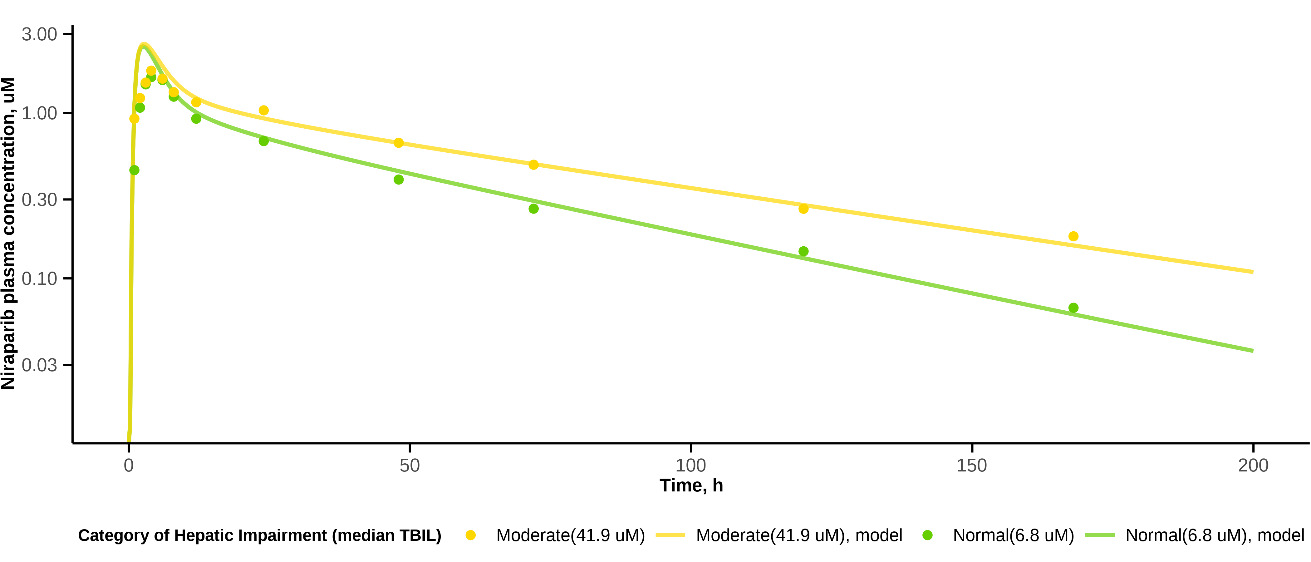


##

## Supplementary Figure S5: Hepatic metabolism parameter CL_spec_ (1/min/μM of enzyme) dependence on total bilirubin (TBIL) level


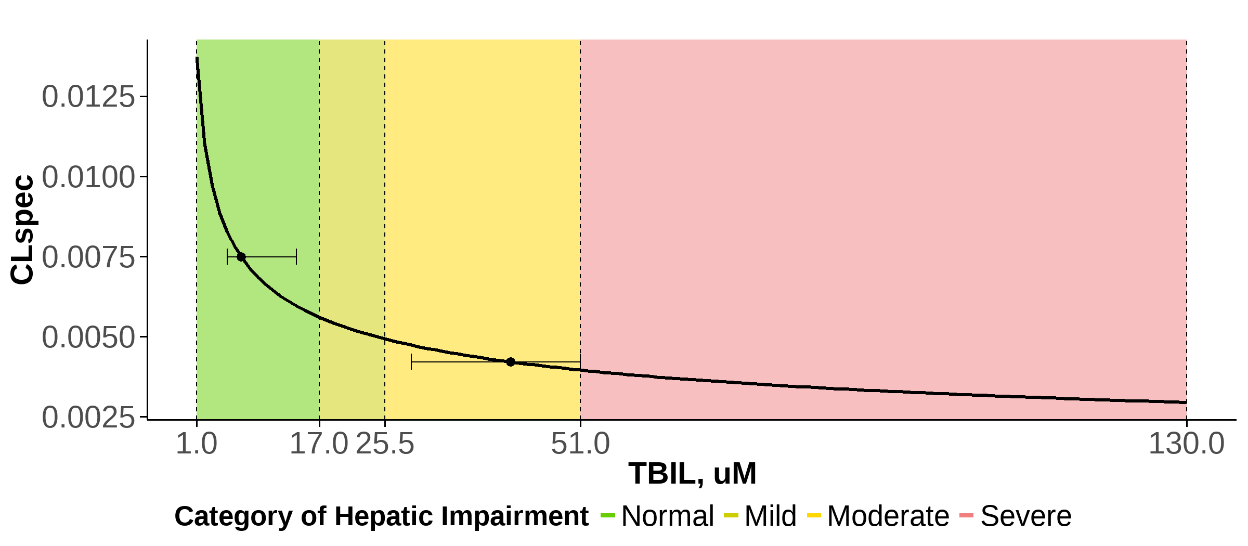


## Supplementary Figure S6: Model-predicted niraparib concentrations in brain compartment


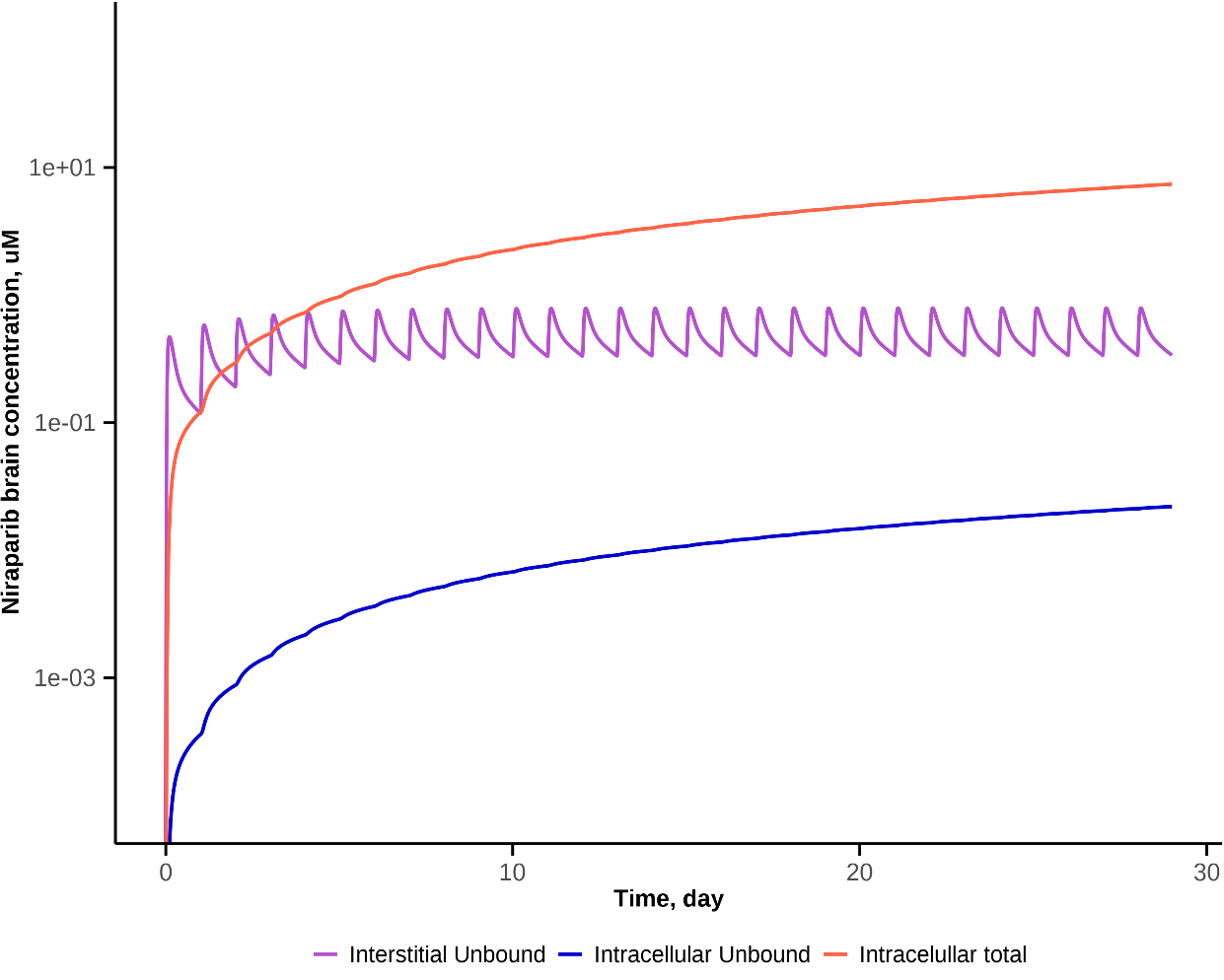


## Supplementary Figure S7: Model-predicted niraparib plasma PK profiles for different hepatic impairment grades

A: Predictions using empirical dependence of CL_spec_ parameter on TBIL level. B: Predictions for individuals with modified physiological parameters values in accordance with the Child-Pugh A, B and C grades of hepatic impairment. In further simulations, the decrease in CES1 abundance in liver was accounted for, in individuals with a CP-C grade.


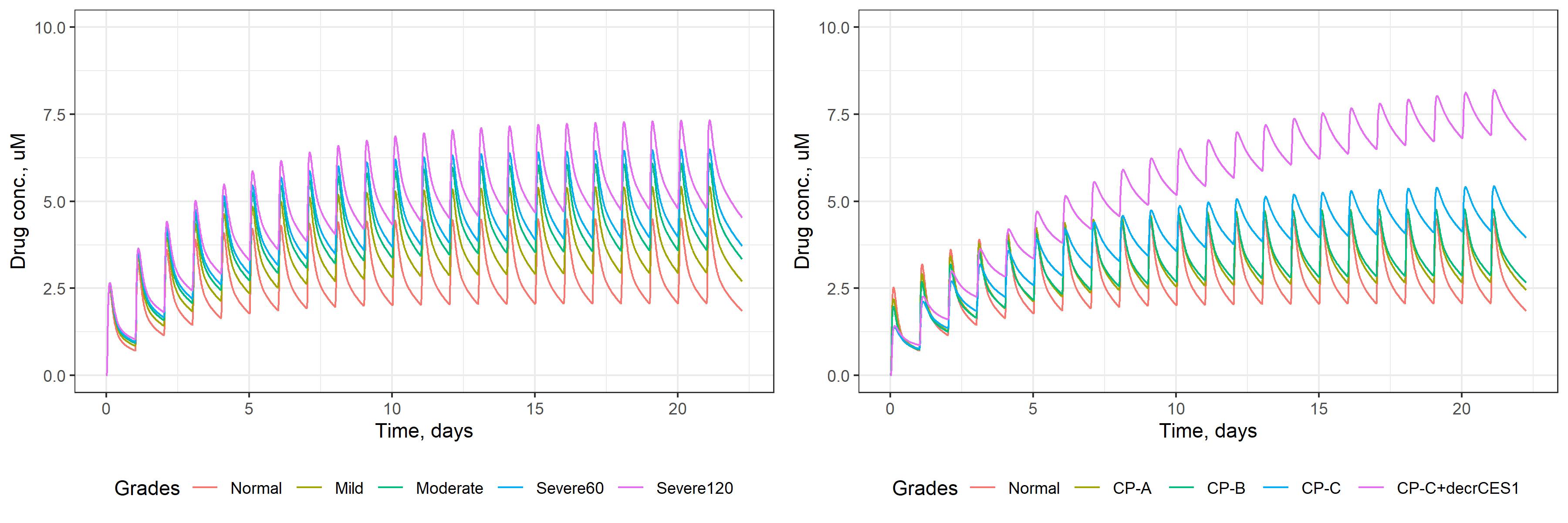


# Supplementary Tables

## Supplementary Table S1: Relative CES1 abundances in various tissues

|  | **Liver** | **Intestine** | **Kidney** | **Heart** | **Lung** |
| --- | --- | --- | --- | --- | --- |
| **Normal hepatic function** (Basit et al., 2020) | | | | | |
| CES1 mean abundance, [pmol/mg S9 protein] | 569.06 | 3.5 | 3.65 | 9.78 | 29.52 |
| MS9 PPGT, [mg S9 protein/gram tissue] | 101.05 | 38.6 | 59.4 | 26.5 | 156.59 |
| CES1 mean abundance,  [pmol/gram tissue] | 57503.513 | 135.1 | 216.81 | 259.17 | 4622.537 |
| Density of tissue, [g/cm^3]  (ICRP 110 - updated Source)  <https://journals.sagepub.com/doi/pdf/10.1177/ANIB_39_2> | 1.05 | 1.04 | 1.05 | 1.05 | 1.06 |
| CES1 mean abundance, [µmol/l tissue] | **60.379** | 0.141 | 0.228 | 0.272 | 4.900 |
| CES1 relative abundance | 1.0000 | 0.0023 | 0.0038 | 0.0045 | 0.0812 |
| **Severe hepatic impairment: CES1 abundance in liver was 30% to normal** (Prasad et al., 2018) | | | | | |
| CES1 mean abundance, [µmol/l tissue] | **18.114** | 0.141 | 0.228 | 0.272 | 4.900 |
| CES1 relative abundance | 1.000 | 0.008 | 0.013 | 0.015 | 0.271 |

## Supplementary Table S2: Summary of Approximate Bayesian Computation Sequential Monte Carlo (ABC-SMC) algorithm parameters

| Iteration | PK observations | Acceptance threshold (ε_t_) | Target number of accepted solutions | Parameter in sampling |
| --- | --- | --- | --- | --- |
| 1 | 210 mg QD | 1 | 50 | logP, pKa, CL_spec_, P_int_, CL_ren_ |
| 2 | 210 mg QD | 0.5 | 50 | -/- |
| 3 | 210 mg QD | 0.2 | 300 | -/- |
| The three parameters were fixed using medians from posterior distributions:  logP= 2.997 and CL_spec_= 0.007489 L/μmol/min | | | | |
| 4 | 210 / 80 / 60 mg QD | 0.20 | 50 | pKa, P_int_, CL_ren_ |
| 5 | 210 / 80 / 60 mg QD | 0.15 | 100 | -/- |
| 6 | 210 / 80 / 60 mg QD | 0.10 | 300 | -/- |
| Medians in final posterior distribution:  pKa = 9.087; P_int_=6.224e-05 dm/min; CL_ren_=3.757e-04 L/min/kg | | | | |

## Supplementary Table S3: Physiological parameter values used in PK-Sim niraparib PBPK model for patients with hepatic impairment

|  | **Normal hepatic function (NHF)** | **Child-Pugh A** | **Child-Pugh B** | **Child-Pugh C** |
| --- | --- | --- | --- | --- |
| Liver volume, l | 1.92 | 1.32 | 1.05 | 0.54 |
| Bone blood flow, l/min | 0.29 | 0.5 | 0.66 | 0.8 |
| Fat blood flow, l/min | 0.5 | 0.85 | 1.12 | 1.37 |
| Gonads blood flow, l/min | 1.18e-3 | 2.00e-3 | 2.64e-3 | 3.21e-3 |
| Heart blood flow, l/min | 0.3 | 0.5 | 0.66 | 0.8 |
| Kidney blood flow, l/min | 1.12 | 0.99 | 0.73 | 0.54 |
| Large intestine blood flow, l/min | 0.3 | 0.12 | 0.11 | 0.01 |
| Liver blood flow, l/min | 0.39 | 0.43 | 0.5 | 0.57 |
| Lung blood flow, l/min | 5.48 | 6.09 | 6.96 | 7.46 |
| Muscle blood flow, l/min | 0.67 | 1.13 | 1.49 | 1.81 |
| Pancreas blood flow, l/min | 0.06 | 0.02 | 0.02 | 2.36e-3 |
| Portal vein blood flow, l/min | 1.21 | 0.49 | 0.44 | 0.05 |
| Skin blood flow, l/min | 0.3 | 0.5 | 0.66 | 0.8 |
| Small intestine blood flow, l/min | 0.65 | 0.26 | 0.23 | 0.03 |
| Spleen blood flow, l/min | 0.15 | 0.06 | 0.05 | 6.02e-3 |
| Stomach blood flow, l/min | 0.06 | 0.02 | 0.02 | 2.36e-3 |
| GFR, ml/min | 107.44 | 107.45 | 75.23 | 38.69 |
| Hematocrit | 0.41 | 0.36 | 0.34 | 0.32 |
| Albumin ratio to NHF / Fraction unbound (plasma) | 1 / 0.17 | 0.81 / 0.2 | 0.68 / 0.23 | 0.5 / 0.29 |

## Supplementary Table S4: Summary of demographics in clinical trials featuring niraparib PK

| Study  (ClinicalTrials.gov ID) | Cohort | Characteristic | | | | | |
| --- | --- | --- | --- | --- | --- | --- | --- |
|  |  | n | Female, n (%) | Age, years | Race, n (%) | | Weight, kg |
|  |  |  |  |  | white | non-white |  |
| (Akce et al., 2021)  (NCT03359850) | Normal hepatic function (V) | 9 | 2(22) | 66(56–76)^†^ | 8(89) | 1(11) | 91.2(19.2)^§^ |
|  | Moderate hepatic impairment (C) | 8 | 4(50) | 65(50–74)^†^ | 8(100) | 0 | 74.9(13.3)^§^ |
| (Mirza et al., 2019)  (NCT02354131) | Overall (V) | 12 | 12(100) | 63.5(51–81)^†^ | - | - | - |
| (Moore et al., 2018)  (NCT01847274) | Fasted (V) | 8 | 8(100) | 64(53–69)^†^ | 6(75) | 2(25) | 79.8(56–108)^†^ |
|  | High-fat meal (V) | 9 | 9(100) | 62(47–68)^†^ | 9(100) | 0 | 66.1(55–114)^†^ |
| (Saad et al., 2021)  (NCT02924766) | Niraparib + APA (V) | 6 | 0(0) | 72(53–81)^†^ | 6(100) | 0 | - |
|  | Niraparib + AAP (V) | 27 | 0(0) | 68(49–82)^†^ | 22(81.5) | 5(18.5) | - |
| (van Andel et al., 2018)  (NCT02476552) | Overall (V) | 6 | 6(100) | 51.5(33–71)^*^ | - | - | 66.7(11.5)^§^ |
| (Yap et al., 2022)  (NCT03307785) | DOS+NIR200 (V) | 16 | 9(56.3) | 63.5(39–85)^†^ | - | - | 82.0(47.7–131.3)^*^ |
|  | DOS+NIR300 (V) | 6 | 2(33.3) | 61.5(40–79)^†^ | - | - | 96.6(78.3–126.2)^*^ |
|  | DOS+NIR200+BEV(V) | 6 | 4(66.7) | 59(37–74)^†^ | - | - | 85.1(59.9–114.1)^*^ |
|  | DOS+NIR300+BEV(V) | 7 | 7(100) | 46(35–66)^†^ | - | - | 88.6(76.4–121.9)^*^ |
| (Sandhu et al., 2013)  (NCT00749502) | Part A (Overall) (V)  (C): 60, 80, 210 mg QD | 60 | 47(78) | 59(35–74)^†^ | - | - | - |
|  | Part B (Overall) (V) | 40 | 22(55) | 56(45–75)^†^ | - | - | - |
| (Yu et al., 2024)  (NCT04577833) | Overall (V) | 136 | 0(0) | 67(50-90)^†^ | 134(99.3) | 1(0.7) | - |
| (Falchook et al., 2024)  (NCT03329001) | BA stage (Overall) (V) | 29 | 18(62.1) | 66(28-88)^†^ | 28(96.6) | 1(3.4) | 78.2(20.32)^§^ |
|  | BE stage (Overall) (V) | 168 | 96(57.1) | 65.5(26-87)^†^ | 125(74.4) | 32(19) | 82.0(20.3)^§^ |

^*^ - Mean (range), ^†^ - Median (range), ^§^ - Mean (SD), C – calibration data, V – validation data.
